# Carbon Disulfide Mediates Socially-Acquired Nicotine Self-Administration

**DOI:** 10.1371/journal.pone.0115222

**Published:** 2014-12-22

**Authors:** Tengfei Wang, Hao Chen

**Affiliations:** Department of Pharmacology, University of Tennessee Health Science Center, Memphis, Tennessee, United States of America; University of Leicester, United Kingdom

## Abstract

The social environment plays a critical role in smoking initiation as well as relapse. We previously reported that rats acquired nicotine self-administration with an olfactogustatory cue only when another rat consuming the same cue was present during self-administration. Because carbon disulfide (CS_2_) mediates social learning of food preference in rodents, we hypothesized that socially acquired nicotine self-administration is also mediated by CS_2_. We tested this hypothesis by placing female adolescent Sprague-Dawley rats in operant chambers equipped with two lickometers. Licking on the active spout meeting a fixed-ratio 10 schedule triggered the concurrent delivery of an i.v. infusion (saline, or 30 µg/kg nicotine, free base) and an appetitive olfactogustatory cue containing CS_2_ (0–500 ppm). Rats that self-administered nicotine with the olfactogustatory cue alone licked less on the active spout than on the inactive spout. Adding CS_2_ to the olfactogustatory cue reversed the preference for the spouts. The group that received 500 ppm CS_2_ and the olfactogustatory cue obtained a significantly greater number of nicotine infusions than other groups. After extinction training, the original self-administration context reinstated nicotine-seeking behavior in all nicotine groups. In addition, in rats that received the olfactogustatory cue and 500 ppm CS_2_ during SA, a social environment where the nicotine-associated olfactory cue is present, induced much stronger drug-seeking behavior compared to a social environment lacking the olfactogustatory cue. These data established that CS_2_ is a critical signal that mediates social learning of nicotine self-administration with olfactogustatory cues in rodents. Additionally, these data showed that the social context can further enhance the drug-seeking behavior induced by the drug-taking environment.

## Introduction

Among the many factors that promote smoking in teenagers, social environment is arguably one of the most critical [1], [2]. Both longitudinal [3] and cross-sectional [4] studies have identified peer smoking as a significant predictor for nicotine dependence. Intriguingly, White, et al. [5], reported that the high concordance in smoking found among monozygotic twins can be sufficiently explained by exposure to similar social environments. Furthermore, social environment also was found to have great impact on smoking cessation [6]. However, the mechanisms underlying the critical influence of social environment are mostly unknown.

Using olfactogustatory stimuli as the contingent sensory cue for i.v. nicotine delivery, we showed the inducing role of social learning in nicotine self-administration (SA) in adolescent rats [7]. In this model, each rat self-administering nicotine was accompanied by a demonstrator rat. These two rats were separated by a divider that allowed orofacial interaction. Licking was used as the operant behavior to deliver an oral olfactogustatory cue contingent with each i.v. nicotine infusion. We found that rats developed conditioned taste aversion to nicotine when the olfactogustatory cue was withheld from the demonstrator rat. In contrast, stable nicotine SA was established when the demonstrator rat had access to the olfactogustatory cue. Because neither the olfactogustatory cue alone nor the demonstrator alone supported nicotine SA, the contingent presentations of the olfactogustatory cue and a signal produced by the demonstrator rat (i.e., a social signal) is required in this model of nicotine SA. The social nature of this model dictated that the behavior of the demonstrator rat affect nicotine intake of the SA rat, which introduces a potential confounding variable should we use this model to study the genetics of nicotine SA [8]. On the other hand, identifying the chemical nature of the social signal could allow us to standardize the social signal across all experimental subjects.

Our nicotine SA protocol parallels the well-established social transmission of food preference paradigm, where interaction with a rat that just consumed flavored food enhanced the preference for that food in naive rats [9]. Galef, et al., established that carbon disulfide (CS_2_), a volatile compound contained in the exhaled breath of rodents was sufficient for social learning of food preference [10]. Because only orofacial interaction is allowed in our model, a volatile compound in exhaled breath is a strong candidate for the social signal. Additionally, the social signal in our model is likely detected at the same time as the olfactogustatory cue from the demonstrator rats. Thus, we tested the hypothesis that contingent presentations of CS_2_ and the olfactogustatory cue supports socially-acquired nicotine SA in the absence of demonstrator rats. Furthermore, because the presence of other smokers in the environment predicted higher likelihood of relapse [11], we further hypothesized that socially-transmitted nicotine cue can enhance the context-induced reinstatement of drug-seeking behavior. Our data supported both hypotheses.

## Materials and Methods

### Animals

Female adolescent Sprague-Dawley rats used for nicotine SA and adult rats used as demonstrators were purchased from Harlan Laboratories (Madison, WI). Adolescent rats were used because the great majority of tobacco use begins during adolescence [12]. Females were used because we found, in one study [8], that female rats were more sensitive to social signals than males. We also reported that adolescent rats acquired nicotine SA more rapidly and attained higher levels of drug intake than adults [13]. Similar to our previous studies [13], [14], rats arrived at our animal facility on approximately postnatal day 31 and received surgery on approximately postnatal day 38. The definition of adolescence in rodents is controversial. According to a conservative perspective in rodents [15], prototypical adolescent changes occur approximately during postnatal day 28–42. Some developmental changes specific to adolescence do persist through PN 55 [15]. Thus, the present experiments were performed within the broadly defined age range of adolescence. Upon arrival, rats were given five to seven days of acclimation to a reversed 12:12 h light–dark cycle (lights off at 9:00 a.m.). Standard rat chow and water were provided *ad libitum*. All rats were group housed with two to four peers throughout the experiments to avoid social isolation. All procedures were conducted in accordance with the NIH Guidelines concerning the Care and Use of Laboratory Animals, and approved by the Institutional Animal Care and Use Committee of the University of Tennessee Health Science Center.

### Experimental treatments

We used 0.4% saccharin and 0.1% unsweetened grape-flavored Kool-Aid (prepared in water) as the olfactogustatory cue. Different concentrations of CS_2_ (10, 100, and 500 ppm, Sigma, St. Louis, MO) were prepared by diluting CS_2_ in the olfactogustatory cue. No solvent was used. The effect of the CS_2_-containing olfactogustatory cue on nicotine SA was tested in the absence of demonstrator rats. The control groups self-administered i.v. nicotine with only the olfactogustatory cue, or only 500 ppm CS_2_ (in water). A third group of control rats self-administered i.v. saline with 500 ppm CS_2_. Contextual cue induced reinstatement was tested in all groups following extinction training.

### Nicotine self-administration

Nicotine SA was conducted according to our published protocol [7] with some modifications. Rats were implanted with jugular catheters constructed using Micro-Renathane tubing (Braintree Scientific Inc., Braintree, MA) under isoflurane anesthesia. The tubing exited from the back of the rat. Ketoprofen (2 mg/kg, s.c.) was given immediately after surgery for postoperative analgesia. After three days of recovery, rats were given access to nicotine SA 3 h per day for 12 days in the dark-phase of the light cycle. The operant chambers were located in sound attenuating chambers and each contained two drinking spouts fitted on the same wall. Two syringe pumps were placed outside of each sound attenuating chamber, one delivered i.v. nicotine through a swivel located on top of the chamber, and the other delivered the olfactogustatory cue to the active spout. The inactive spout contained no solution. Each spout was connected to a contact lickometer controller allowing the number and the timing of licks to be recorded. SA was conducted using a fixed-ratio 10 schedule with 20 s timeout period (FR10TO20). Thus, 10 licks on the active spout activated the simultaneous delivery of a 60 µl olfactogustatory cue, and an i.v. infusion (nicotine free base, 30 µg/kg or saline). The olfactogustatory solution contained saccharin (0.4%) and unsweetened grape Kool-Aid (0.1%) as well as different concentrations of CS_2_ (0–500 ppm). A previous report [16] showed that oral CS_2_ exposure at 100–253 mg/kg/day caused no adverse effects on systemic, neurological and developmental systems in rats. Licks on the inactive spout had no programmed consequence. Licks during the timeout period had no consequences but were recorded. No audio or visual cue was used. Rats were not food or water deprived. Nor did rats receive operant training or priming nicotine injections prior to the initiation of the SA sessions. The patency of the jugular catheters was tested using a fast acting anesthetic, methohexital (0.2 ml, 10 mg/ml), at the end of the 12 SA sessions for each rat. Rats without functional catheters were excluded from the analysis.

### Extinction and context-induced reinstatement

Context extinction training was conducted after 12 SA sessions. The context extinction chambers were different from the SA operant chamber in many aspects, including the floor, distinct audiovisual cues and novel odor [17]. There were two clean dry spouts in the chamber. The number of licks were recorded but had no programmed consequence. Because most of the licking during extinction training occurs early during the session, we reduced the time of extinction to one hour per session. Extinction sessions were conducted daily until the number of licks on the “active” spout was reduced to less than 50 for two consecutive daily sessions.

**Figure 1:**
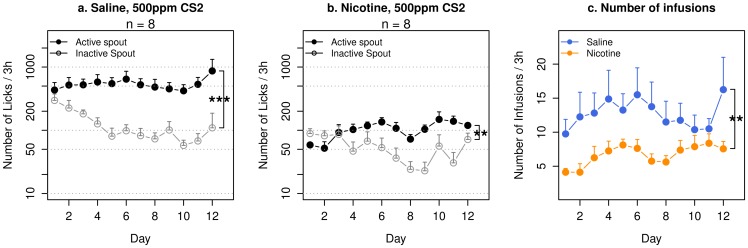
Nicotine self-administration with CS_2_ in adolescent rats. Adolescent female Sprague-Dawley rats implanted with jugular catheters were placed in operant chambers equipped with two lickometers. Licking on the active spout that met a fixed-ratio 10 reinforcement schedule triggered contingent delivery of oral CS_2_ (500 ppm, dissolved in water) and i.v. saline (a) or i.v. nicotine (b). No olfactogustatory cue was used. Both groups showed preference for the active spout. The average number of infusions (c) was 12.9±1.9 for rats that self-administered saline, and 6.7±0.4 for rats that self-administered nicotine. **: p<0.01; ***: p<0.001, repeated measures ANOVA.

Two reinstatement tests (one session per day, each session was one hour) were conducted once the extinction criterion was met to examine whether the presence of socially-transmitted nicotine cue could enhance context-induced nicotine seeking behavior. During these tests, rats were placed in the original SA chambers but licking on the spouts had no consequence. Because it is likely appetitive, CS_2_ was not delivered by the licking spout (Fig. 1a). Instead, a randomly-selected, unfamiliar demonstrator rat was used to provide the social environment. A perforated divider was used to separate the demonstrator and the SA rat. In one of the sessions the demonstrator rat had access to the olfactogustatory cue, thus providing an inducing social environment (ISE). In the other session, the demonstrator rat did not have access to the olfactogustatory cue, providing a neutral social environment (NSE). The order of social environment was counterbalanced between rats within the same treatment group.

### Statistical analysis

The number of licks were converted to log scale so that the data fit a normal distribution. The effects of CS_2_ and nicotine on the number of licks and infusions were analyzed using repeated measures ANOVA. Post-hoc tests were conducted using the Tukey HSD procedure when necessary. Spout and session were treated as within-subject variables, while CS_2_ concentration and i.v. treatment were between subject variables. Data were presented as mean ± SEM. Statistical significance was assigned when p<0.05. All statistical analyses were conducted using the R statistical language.

## Results

### Saline or nicotine self-administration with CS_2_ as the cue

Rats that received contingent oral CS_2_ (500 ppm) with i.v. saline (Fig. 1a) showed a preference for the active spout (F_1,7_ = 102.4, p<0.001). The number of licks on the active spout did not change significantly across the sessions (F_11,77_ = 0.5, p>0.05), neither did the number of infusions change (Fig. 1c. F_11,77_ = 1.4, p>0.05). There was a significant interaction between spout and session (F_11,77_ = 2.4, p<0.05). On average, 12.9±1.9 infusions were obtained. These data suggested that CS_2_ is appetitive.

Rats that received contingent oral CS_2_ (500 ppm) with i.v. nicotine (Fig. 1b) showed a preference for the active spout (F_1,7_ = 16.4, p<0.01). The number of licks on the active spout increased significantly across the sessions (F_11,77_ = 2.4, p<0.05). However, the number of infusions did not change significantly (Fig. 1c. F_11,77_ = 1.4, p>0.05). There was a significant interaction between spout and session (F_11,77_ = 9.0, p<0.01). On average, 6.7±0.4 infusions were obtained. These data suggested that CS_2_ supported nicotine SA.

Rats that received i.v. saline emitted significantly more licks on the active spout compared to rats that received i.v. nicotine (Fig. 1a, 1b, F_1,13_ = 40.0, p<0.001), and obtained significantly more infusions (Fig. 1c. F_1,13_ = 14.8, p<0.01), suggesting that nicotine is potentially aversive.

### Nicotine self-administration with an olfactogustatory cue and different CS_2_ concentrations

**Figure 2:**
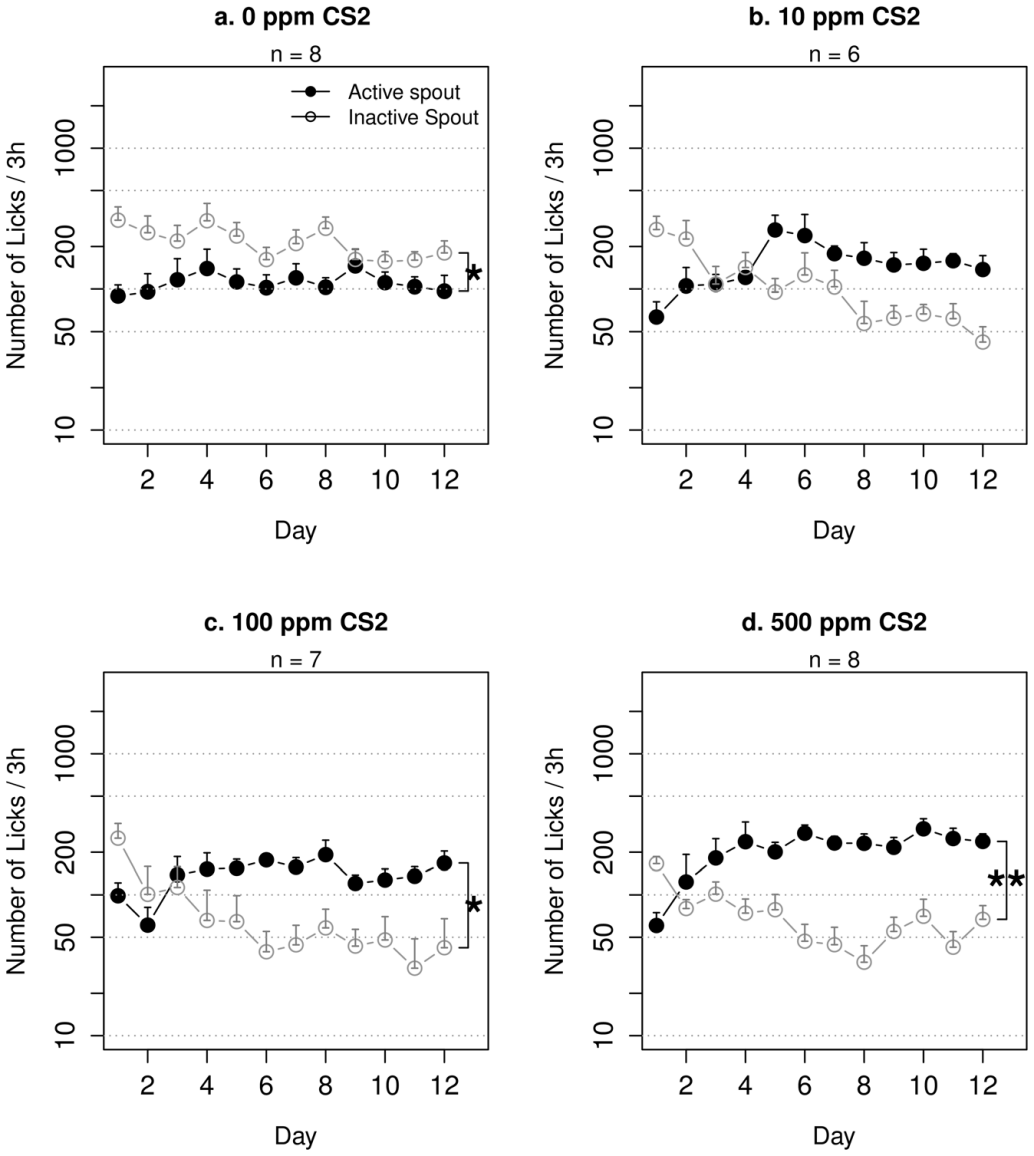
Nicotine self-administration with an olfactogustatory cue and CS_2_ in adolescent rats. Adolescent rats self-administered i.v. nicotine with contingent olfactogustatory cue containing different concentrations of CS_2_. Rats that received only the olfactogustatory cue (a) licked less on the active spout. With increasing concentration of CS_2_ included in the olfactogustatory cue, the number of licks on the active spout took fewer sessions to surpass that on the inactive spouts (10 ppm: 4 sessions, 100 ppm: 3 sessions, 500 ppm: 1 session). *: p<0.05; **: p<0.01, repeated measures ANOVA.

As expected based on our previous studies [7], rats that self-administered i.v. nicotine with a contingent oral olfactogustatory cue without CS_2_ (Fig. 2a) emitted fewer licks on the active spout compared to the inactive spout (F_1,7_ = 7.0, p<0.05), suggesting that contingent olfactogustatory cue was associated with the aversive effect of nicotine. The number of licks did not change over the sessions (F_11,77_ = 0.4, p>0.05), suggesting extending the training did not change the overall subjective value of the stimuli (i.e., nicotine and the olfactogustatory cue).

Rats that received nicotine SA with contingent olfactogustatory cue and 10 ppm CS_2_ (Fig. 2b) did not show preference for either spout (F_1,5_ = 3.2, p>0.05). However, the number of licks on the active spout significantly increased across the sessions (F_11,55_ = 2.4, p<0.05). There was a significant interaction between the effect of spout and session (F_11,55_ = 7.5, p<0.001). The average number of licks on the active spout exceeded that of the inactive spout starting from day 5 and remained greater throughout the rest of the training sessions, suggesting that the inclusion of CS_2_ gradually changed the affective value of the stimuli (i.e., nicotine, the olfactogustatory cue, and CS_2_)

Rats that received nicotine SA with contingent olfactogustatory cue and 100 ppm CS_2_ (Fig. 2c) showed significant preference for the active spout (F_1,6_ = 9.5, p<0.05), suggesting that the overall stimuli was appetitive. The interaction between spout and session was statistically significant (F_11,66_ = 5.3, p<0.001) The number of licks on the active spout increased significantly across the sessions (F_11,66_ = 2.8, p<0.01). The average number of licks on the active spout exceeded that of the inactive spout starting from day 4 and remained greater throughout the rest of the training sessions.

Rats that received nicotine SA with contingent olfactogustatory cue and 500 ppm CS_2_ (Fig. 2d) showed significant preference for the active spout (F_1,7_ = 27.4, p<0.01). The number of licks on the active spout increased significantly across the sessions (F_11,77_ = 5.2, p<0.001). The interaction between spout and session was significant (F_11,77_ = 10.4, p<0.001). The average number of licks on the active spout exceeded that of the inactive spout starting from day 2 and remained greater throughout the rest of the training sessions.

**Figure 3:**
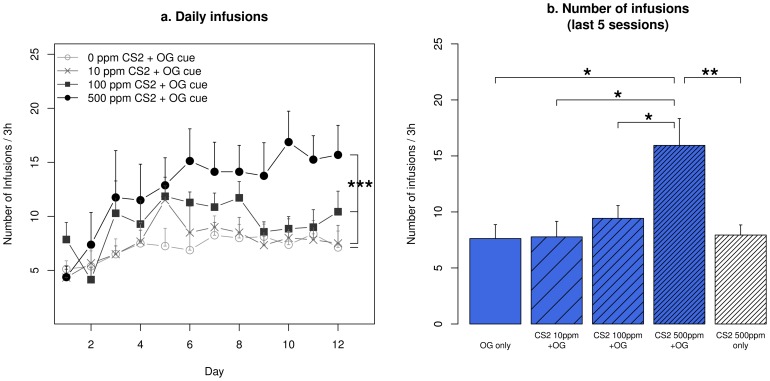
Number of infusions obtained by rats self-administering nicotine with an olfactogustatory cue and different concentrations of CS_2_. (a) The number of daily infusions obtained by rats that received 500 ppm CS_2_ was significantly higher than all other groups. (b) The average number of infusions obtained during the last five sessions was compared between different oral cues. * p<0.05, Tukey HSD; ** p<0.01, Tukey HSD; ***: p<0.001, Repeated measures ANOVA.

In Fig. 3a. we compare the number of nicotine infusions obtained by rats receiving nicotine SA with olfactogustatory cue and different concentrations of CS_2_. Repeated measures ANOVA found that the number of infusions increased significantly for all groups (F_11,319_ = 7.1, p<0.001). Tukey HSD post-hoc test showed rats that received 500 ppm CS_2_ obtained significantly more infusions compared to all three other groups (p<0.001, p<0.001, and p<0.01 for 0, 10, and 100 ppm CS_2_, respectively). Fig. 3b summarizes the number of infusions for the last five days, when nicotine infusion was stable. Rats that received 500 ppm CS_2_ and olfactogustatory cue obtained significantly more infusions compared to those that received the olfactogustatory cue with lower CS_2_ concentrations or no CS_2_ (p<0.05 for all), or 500 ppm CS_2_ without olfactogustatory cue (p<0.01).

### Context-induced drug-seeking behavior

**Figure 4:**
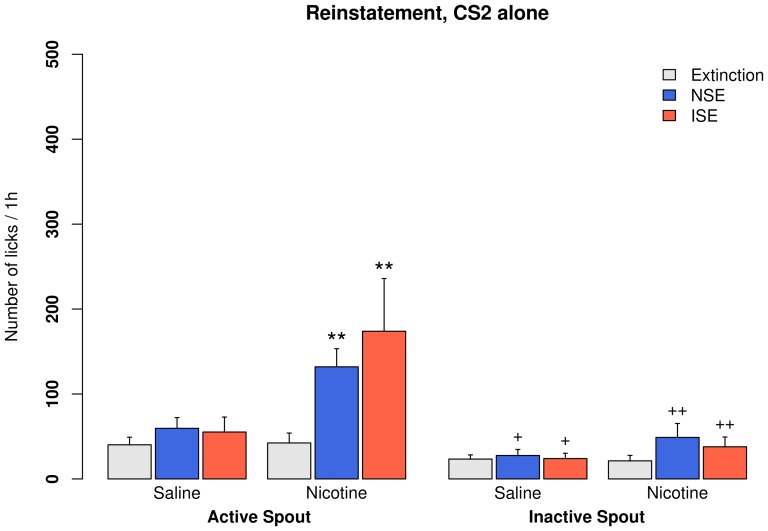
Context-induced drug-seeking in rats self-administering saline or nicotine with CS_2_. Extinction training was conducted in operant chambers different from those used in the self-administration sessions. Two reinstatement tests were conducted consecutively in the presence of a demonstrator rat. The demonstrator rat either provided a neutral social environment (NSE, i.e., did not have access to the olfactogustatory cue), or an inducing social environment (ISE, i.e., consuming olfactogustatory olfactogustatory cue). The sequence of tests were counterbalanced between rats. **: p<0.01, compared to extinction; +: p<0.05, compared to the active spouts; ++: p<0.01, compared to the active spouts.

**Figure 5:**
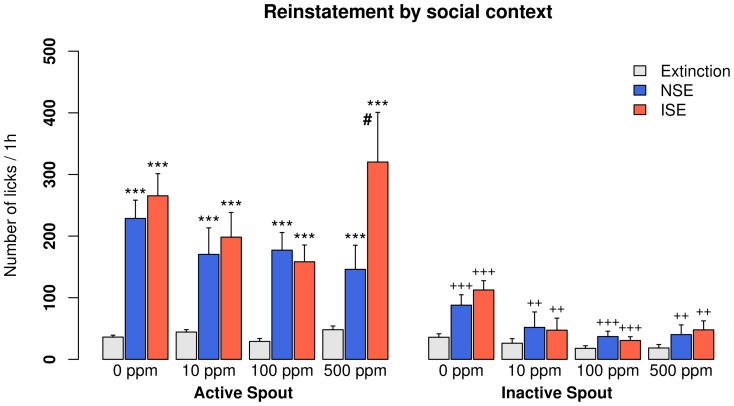
Context-induced drug-seeking in rats self-administering nicotine with an olfactogustatory cue and different concentrations of CS_2_. The nicotine self-administration environment induced strong drug-seeking behavior in all nicotine groups. In rats that received 500 ppm CS_2_ during self-administration, the inducing social environment induced a significantly greater amount of drug-seeking behavior compared to the neutral social environment. ***: p<0.001 compared to extinction; ++: p<0.01 compared to the active spouts; +++: p<0.001 compared to the active spouts; #: p<0.05 compared to NSE. NSE: the neutral social environment; ISE: the inducing social environment.

Most rats reached the extinction criteria in less than five sessions. The main effects of re-exposing to the SA context, the types of social environment (ISE vs. NSE), and the spout (active vs. inactive) are shown in Table 1. No statistically significant interaction was found. Overall, the original SA context did not change the number of active licks during reinstatement tests in rats that received i.v. saline with a CS_2_ cue (Fig. 4). In contrast, context-induced drug-seeking behavior was seen in all groups that received i.v. nicotine, regardless of the sensory cues they received (Figs. 4 and 5). The effect of social context was only significant in the group that received 500 ppm CS_2_, where the number of active licks was 320±80.7 in the ISE and 146±39.2 in the NSE.

**Table 1:** Statistical results for context-induced reinstatement.

OG cue	CS_2_ (ppm)	i.v. Drug	Reinstatement	Social context	Spout
No	500	Saline	F_2,16_ = 0.9, p>0.05	p>0.05	F_1,6_ = 18.5, p<0.05
No	500	Nicotine	F_2,16_ = 2.2, p<0.01	p>0.05	F_1,6_ = 33.1, p<0.01
Yes	0	Nicotine	F_2,19_ = 53.3, p<0.001	p>0.05	F_1,8_ = 55.0, p<0.001
Yes	10	Nicotine	F_2,13_ = 18.5, p<0.001	p>0.05	F_1,5_ = 24.5, p<0.01
Yes	100	Nicotine	F_2,16_ = 48.2, p<0.001	p>0.05	F_1,7_ = 79.5, p<0.001
Yes	500	Nicotine	F_2,16_ = 20.1, p<0.001	p<0.05	F_1,6_ = 16.7, p<0.01

Two reinstatement tests were conducted consecutively in the presence of demonstrator rats that provided either a neutral social environment or an inducing social environment. Repeated measures ANOVA was used to compare the number of licks on the active spout between extinction and reinstatement, the number of licks on the active spout between the two social contexts, and the number of licks on the active vs. the inactive spout during reinstatement tests.

## Discussion

We showed that a contingent olfactogustatory cue was associated with the aversive effect of self-administered nicotine. However, the addition of CS_2_ to the olfactogustatory cue supported nicotine SA. Among the three groups of rats that self-administered nicotine with an olfactogustatory cue and CS_2_, both the 100 and 500 ppm CS_2_ groups showed preference for the active spout. After extinction training, the original SA context reinstated nicotine-seeking behavior in all groups that self-administered nicotine. Further, in rats that self-administered nicotine with 500 ppm CS_2_ and an olfactogustatory cue, the socially-transmitted nicotine-associated odor cue increased drug-seeking behavior above that induced by a neutral social environment.

Many clinical studies have shown that nicotine has few positive affective effects. Rather, aversive effects, such as nausea, dizziness, coughing, and headache are much more common [12], [18]. In spite of these negative effects, approximately 40% of teenagers that experiment with cigarettes become regular smokers [19]. One critical factor that has large influence on smoking initiation is the social environment [5], [20], [21]. We previously reported a model of nicotine SA in adolescent rats, where the presence of a demonstrator rat that carried the nicotine-associated olfactogustatory cue was a determining factor for the acquisition of SA [7]. The data presented here identified CS_2_ as the social signal that mediated the effect of the demonstrator rats.

CS_2_ is produced by respiratory tract [22] and gut [23] bacteria and is present in the breath of rodents and humans [24]. A recent study showed that a specialized type of olfactory sensory neuron that expresses the receptor guanylyl cyclase type D (GC-D) is required for the detection of CS_2_. Mice with mutations in the genes of this pathway are deficient in social learning [25]. However, it is unlikely that CS_2_ is a social signal that facilitates smoking among teenagers, because social learning in primates and humans requires much more complex cognitive functions [26]. On the other hand, we argue that despite the vast difference in the signals involved in social learning, the central processing of such signals is, to some extent, conserved. One example is the neuropeptide oxytocin, which mediates a variety of social behaviors in both humans and rodents, including social learning [27], [28]. In a separate study, we have begun looking at the role of this neuropeptide in nicotine SA using our model. Therefore, our model has the potential to reveal mechanisms of social learning critical for smoking in humans. One practical advantage of using CS_2_ is that it allows the amount of social signal to be standardized. Therefore, it eliminates the variation caused by the different behaviors among demonstrators [8].

One key finding from these experiments is that CS_2_ reversed the preference for the spouts in rats self-administering nicotine. We have found that the ratio of licks on the active/inactive spout has a strong correlation (r = 0.8, p<0.001) with the size of lick clusters (Wang and coworkers, manuscript under review). The size of lick clusters reflects the subjective value of oral stimuli [29], with appetitive stimuli producing larger lick clusters. Therefore, a greater number of licks on the active spout than on the inactive spout indicates that a stimulus is appetitive. Fig. 1a showed that rats preferred the active spout when CS_2_ alone was used as the sensory cue for i.v. saline, which suggested that CS_2_ *per se* was appetitive. This was in agreement with findings that social environments are rewarding [30]. Fig. 1b showed that rats that self-administered nicotine with CS_2_ alone also preferred the active spout, suggesting that the combined stimuli provided by CS_2_ and nicotine was appetitive.

In contrast to Fig 1b, our previous study (see Fig. 6 in [7]) found that rats that self-administered nicotine accompanied by demonstrators without access to the odor cue did not prefer the active spout. These two experiments were similar in that rats self-administered nicotine in an environment where CS_2_ was present (contained in the olfactogustatory cue in this experiment and from live companion rats in our previous experiment). However, there are two major experimental design differences: 1) Rats in Fig. 1b did not receive contingent olfactogustatory cue, while the SA rats in our previous study received contingent taste cue; 2) Rats in Fig. 1b received CS_2_ as a discrete cue contingent with the delivery of nicotine, while in our previous study the demonstrator was part of the context. Therefore, a likely explanation for these data is that nicotine produces both rewarding and aversive effects. The overall subjective effect of nicotine is determined by the sensory stimuli contingently presented with nicotine. Odor and taste cues are associated with nicotine induced aversion, even when the cues are highly appetitive [7]. Social cues however, are associated with nicotine reward (Fig. 1b). Lastly, the odor cue needed to be socially-transmitted [7], or presented together with CS_2_ (Fig. 2) to overcome the conditioned taste and odor aversion produced by nicotine.

Fig. 2 showed that the number of inactive licks were higher than the active ones throughout the 12 sessions in the group that received nicotine SA with an olfactogustatory cue but not CS_2_. However, with increasing CS_2_ concentrations, the number of active licks took progressively fewer sessions to surpass that of the inactive licks. Because CS_2_ *per se* is appetitive (Fig. 1a), the behavioral changes could result from the enhanced reward value of CS_2_, the interaction between the reward values of nicotine and CS_2_, or a weakened association between the aversive effect of nicotine and the olfactogustatory cue.. Our current data cannot differentiate between these alternative mechanisms. Although it is difficult to equate the CS_2_ concentrations with live rats, the data shown in Fig. 3 indicated that rats that received 10–100 ppm obtained approximately the same amounts of nicotine as those accompanied by randomly selected unfamiliar demonstrators [7]. We also reported that familiar demonstrators facilitated an enhanced nicotine intake [7], which was similar to those obtained by rats that received 500 ppm CS_2_ in this study.

Clinical studies have found that exposure to a smoking-related environment elicits a robust craving to smoke [31]. The presence of other smokers in the environment predicted a higher likelihood of relapse [11], [32]. Therefore, we tested whether socially-transmitted nicotine cue can enhance context-induced reinstatement of drug-seeking behavior. We have previously shown [8] that re-exposing rats that acquired nicotine SA in a social setting to the nicotine-taking environment induced strong reinstatement behavior. Additionally, the amount of social interaction during reinstatement was a significant predictor of reinstatement. However, because reinstatement behavior was only tested in an environment containing demonstrators actively consuming the olfactogustatory cue in that study, the role of socially-transmitted signal was not dissociable from the overall environment. In the current study, we clarified the role of socially-transmitted odor cue during the reinstatement test. We did not contingently present CS_2_ during the reinstatement tests because CS_2_ is likely rewarding (Fig. 1a). Instead, we provided demonstrator rats in the nicotine-taking environment during two consecutive reinstatement tests, where the demonstrators either did or did not have access to the olfactogustatory cue. Figs. 4 and 5 showed that the SA environment induced drug-seeking behavior in all groups that self-administered nicotine. Furthermore, in rats that received the olfactogustatory cue and 500 ppm CS_2_ during SA, a social environment where nicotine associated olfactory cue is socially-transmitted (i.e., ISE) induced much stronger drug-seeking behavior compared to a social environment lacking that olfactogustatory cue (NSE). Therefore, these data showed that socially-transmitted nicotine-associated odor cue enhanced drug-seeking behavior.

In summary, we have established a nicotine SA model in adolescent rats where stable nicotine intake is enabled by contingent presentations of CS_2_ and an olfactogustatory cue. In addition, socially-transmitted nicotine-associated odor cue enhanced drug-seeking behavior induced by the drug-taking context.
